# In Search of a Double Perovskite in the Phase Triangle of Bromides CsBr-CuBr-InBr_3_

**DOI:** 10.3390/ma16103744

**Published:** 2023-05-15

**Authors:** Rustam K. Kamilov, Jahongir Z. Yuldoshev, Alexander V. Knotko, Anastasia V. Grigorieva

**Affiliations:** 1 Department of Material Science, Lomonosov Moscow State University, 119991 Moscow, Russia; 2 Department of Chemistry, Lomonosov Moscow State University, 119991 Moscow, Russia

**Keywords:** double perovskite bromide, melt crystallization, solid state sintering, phase equilibria

## Abstract

New bromide compounds A_2_B^I^B^III^Br_6_ with a double perovskite structure provide variety and flexibility of optoelectronic properties, and some of them are of poor toxicity in comparison with such popular lead halides. The promising compound with a double perovskite structure was proposed recently for the ternary system of CsBr-CuBr-InBr_3_. Analysis of phase equilibria in the CsBr-CuBr-InBr_3_ ternary system showed stability of the quasi-binary section of CsCu_2_Br_3_–Cs_3_In_2_Br_9_. Formation of the estimated phase Cs_2_CuInBr_6_ by melt crystallization or solid-state sintering was not observed, most likely, as a result of higher thermodynamic stability of binary bromides CsCu_2_Br_3_ and Cs_3_In_2_Br_9_. The existence of three quasi-binary sections was observed, while no ternary bromide compounds were found.

## 1. Introduction

In recent decades, solutions for “green” energy technologies have been of great importance [1,2]. The photovoltaic systems of metal halide perovskites have been in focus since the report of Kojima et al. in 2006 when he used a hybrid lead halide perovskite CH_3_NH_3_PbI_3_ in a photovoltaic cell with a structure similar to dye-sensitized solar cells, and later in 2009, an efficiency of 3.8% was published [3,4]. Developed by Kim et al. and Li et al. in 2012, MAPbI_3_ perovskite solid-state solar cells with an efficiency of 9.7% and 10.9%, respectively, became a breakthrough in photovoltaics [5,6]. Every year, the efficiency of lead-based perovskite solar cells increases, making them promising materials not as photovoltaics only. The efficiency of perovskite solar cells currently exceeds 25% [7]. The almost perfect optoelectronic properties of lead halides arose from a forward bandgap, high absorption coefficient in a visible range, the long diffusion length of a carrier and excellent carrier mobility, well-balanced electron and hole mobility, high dielectric constant, and low halide binding energy excitons. Perovskite-like inorganic halides have demonstrated their perspectives in optoelectronics as emitters, light-emitting diodes (LEDs) [8,9,10], lasers [11], photodetectors [12,13], etc. It is a big challenge to investigate photoluminescent and transport characteristics, predict a band structure and simulate physicochemical properties for new complex halides which have not been synthesized yet. Many works are focused on 1D or 2D hybrid halides, such as bismuth- and copper-based complex halides [14,15,16,17]. At the same time, inorganic halides are still attractive because of their higher thermal and photostability. Despite inorganic halides, a group of “double perovskite” halides belong to the most promising materials.

At the same time, a number of disadvantages of efficient perovskite or perovskite-like halides are chemical toxicity [18,19], poor temperature stability [20,21], photodegradation [21,22], hydrolysis by traces of water and oxygen [22], and also self-decomposition [23]. The crystal structure of double perovskite A_2_B^I^B^III^X_6_ can be formed by a heterovalent substitution of two bivalent Pb^2+^ cations in the octahedral positions with a pair of non-toxic M^+^ and M^3+^ metal cations. This makes such an alternative promising for the implementation of optoelectronic or perovskite photovoltaic devices. Recent theoretical simulations show that the crystal structure of a double perovskite A_2_B^I^B^III^X_6_ provides the possibility of easier substitution of cations in the B-position and a number of inorganic and organic cations in the A-position in comparison with classic perovskite halides ABX_3_ [24,25,26]. Additionally, variations in the halide composition in X-positions make it possible to obtain new thermodynamically stable halides with even four- or more cations. The predicted double perovskite materials are attractive as lead-free materials for perovskite solar cells, light-emitting diodes, and optical switchers.

Recently, a number of double perovskites, such as Cs_2_AgBiX_6_ (X = Cl^−^, Br^−^) and Cs_2_AgInCl_6_, were obtained experimentally [24,27,28]. However, many more double perovskite compounds have been predicted to exist but were not obtained yet. Some of them have a direct band gap and high carrier mobility, which is optimal for both LED and photovoltaic applications.

According to Zhao et al. [29] and Li et al. [30], the Cs_2_CuInBr_6_ phase is stable. The compound is a semiconductor with a bandgap of ~0.6 eV and excellent mobility of charge carriers. Such a complex of parameters is attractive for electronic devices [31]. According to simulations reported by Xiao et al. [32], in the ternary system CsBr-CuBr-InBr_3_ the binary bromide Cs_2_CuInBr_6_ with a double perovskite structure decomposes to binary bromides CsCu_2_Br_3_ and Cs_3_In_2_Br_9_. In the present manuscript, we present the most recent experimental results on the investigation of crystallization processes in the ternary system of simple bromides CsBr-CuBr-InBr_3_ in a temperature range of 300–650 °C. 

## 2. Experimental

### 2.1. Methods of Analysis

X-ray diffraction data were obtained using a diffractometer Rigaku 2500 D-max (Rigaku, Tokyo, Japan) with a rotating copper anode (λ = 1.5418 Å). Diffractograms were registered in the 2θ range of 10–80°, and a step was 0.02°. The XRD data were processed using software Jana2006 (ECA-SIG#3/Institute of Physics, Prague, Czech Republic) software.

The Raman spectra of the samples were collected using an InVia Raman Microscope spectrometer (Renishaw, New Mills, UK) equipped with an argon laser λ = 532 nm (power of 20 mW, 5% of the total intensity). All spectra were obtained using a 50× objective lens by 100-fold signal accumulation, excitation time 1 s. As a standard sample for the zero-calibration, we used a plate of (100)-oriented silicon single-crystal plane.

The chemical composition of the samples and the mapping images were examined by the energy dispersive X-ray spectroscopy (EDS) using a Leo Supra 50 VP microscope (LEO Carl Zeiss SMT Ltd., Oberkochen, Germany) with an X/MAX X-ray energy dispersive detector (Oxford Instruments, High Wycombe, UK). In most experiments, an electron accelerating voltage was 20 kV.

Differential scanning calorimetry (DSC) measurements were performed in a temperature range of 200–400 °C using a differential scanning calorimeter DSC 404 C Pegasus (NETZSCH, Selb, Germany). All measurements were carried out in an argon atmosphere to avoid redox processes in materials.

### 2.2. Synthesis of Compounds in CsBr-CuBr-InBr_3_ Triangle

Ampoule synthesis served as the main approach in the sample preparation. Various compositions of simple and binary bromides were taken as precursors. Cesium bromide was taken after Sigma-Aldrich (99.999%). Copper (I) bromide was synthesized by the solution method via a redox reaction, according to Equation (1). Sodium sulfite (“Rushim”, pure) with sulfuric acid (“Sigma Tech”, pure) as a source of sulfur oxide (IV) is also used. A strong flow of pure SO_2_ was passed into a solution of appropriate amounts of pure copper (II) sulfate pentahydrate (“Rushim”, pure) and potassium bromide (“Reahim”, extra pure) in distilled water at moderate heating (T = 60 °C) for 2 h. In this case, it is a light precipitate in the form of thin yellowish-white crystals. Carefully protecting the precipitate from the action of daylight, it is filtered off 5–7 times, placed in boiled distilled water containing SO_2_, and filtered again. After that, the precipitate was washed with 96% alcohol and then treated in a nitrogen atmosphere at 60 °C.

2CuSO_4_∙5H_2_O + 2KBr + SO_2_ = 2CuBr + 2H_2_SO_4_ + K_2_SO_4_ + 8H_2_O (1)

Indium bromide InBr_3_ was prepared by the wet-chemistry method using elementary indium (“RedkyMetal.RF”, 99.999%) and hydrobromic acid HBr (“Reahim”, analytical grade) as precursors [33,34,35].

The ampoules syntheses were carried out in closed quartz ampoules according to the technique discussed elsewhere [35]. The total weight of each sample was 2 g. The ampoules were sealed after their evacuation (0.072 bar). 

**Figure 1:**
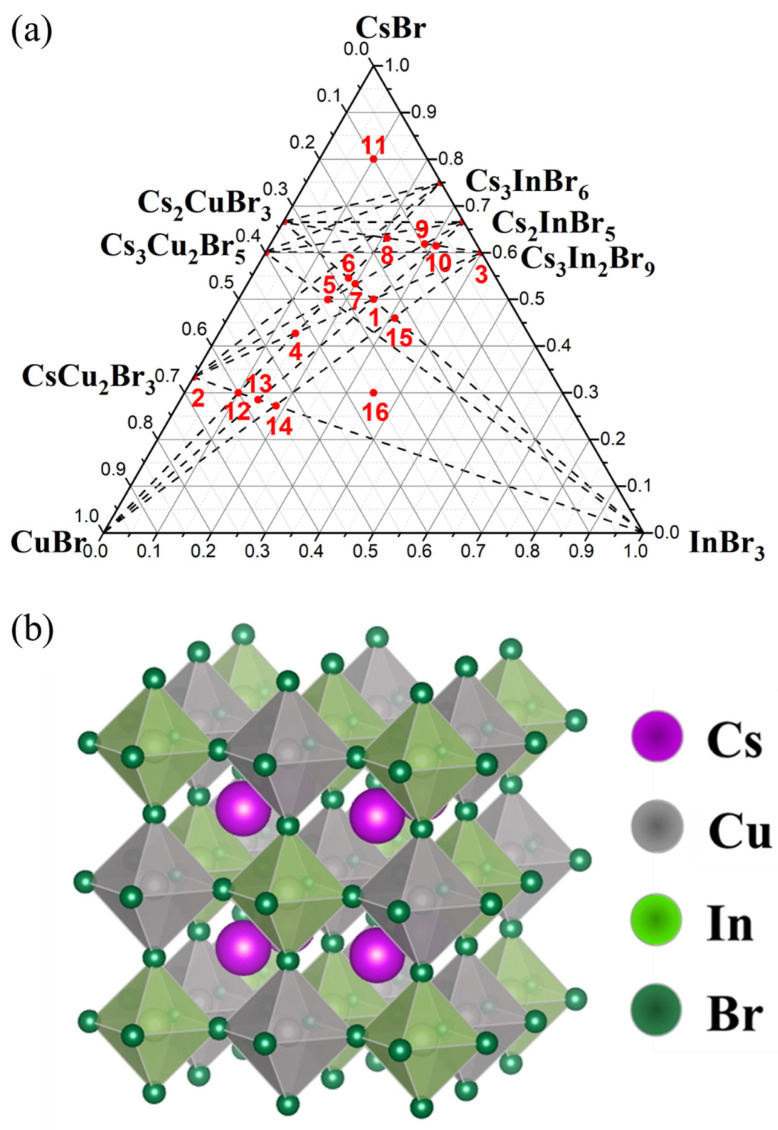
Gibbs triangle for the (**a**) CsBr-CuBr-InBr_3_ with selected compositions marked as points, and (**b**) a 2 × 2 unit cell of the double perovskite Cs_2_CuInBr_6_.

A number of compositions in the CsBr-CuBr-InBr_3_ phase triangle of bromides were taken for analysis of phase equilibria, and the availability of a “double perovskite” was predicted theoretically (Figure 1a). The composition ‘1’ corresponds to the “double perovskite” Cs_2_CuInBr_6_ phase, according to Ref. [29]. The double perovskite Cs_2_CuInBr_6_ has a cubic structure with a space group Fm3̅m with a lattice constant of 10.9 Å. The unit cell of Cs_2_CuInBr_6_ consists of CuBr_6_ and InBr_6_ octahedra alternating along all three directions. And the B-site cations Cu^+^ and In^3+^ form an ordered structure of double perovskite, as shown in Figure 1b. 

The weights of precursors, including cesium bromide, copper(I) bromide, and indium(III) bromide, per 1 g of the resulting sample weights are given in Table 1. The measurements were carried out using a semi-micro analytical balance GR-202 (A&D Company, Limited, Tokyo, Japan) at room temperature.

Samples of theoretically predicted composition Cs_2_CuInBr_6_ were synthesized by the solid-phase or heterophase ampoule methods. The annealings were carried out at T = 300–650 °C, and the duration was 96 h in most of the experiments, according to recent results reported by Kamilov et al. for a similar ternary system [35].

## 3. Results and Discussion

Three binary systems of single bromides are the sides of the phase triangle CsBr-CuBr-InBr_3_. In the CsBr–CuBr binary system, there are three phases–Cs_2_CuBr_3_ and CsCu_2_Br_3_ [36], Cs_3_Cu_2_Br_5_ [37], and in the CsBr–InBr_3_ system, three binary bromides, namely, Cs_3_In_2_Br_9_ [38], Cs_3_InBr_6_, and Cs_2_InBr_5_ [39] could be formed. No binary bromides are predicted for the binary CuBr–InBr_3_ system. 

The “double perovskite” composition (point ‘1’ in the phase triangle) in CsBr-CuBr-InBr_3_ Gibbs’s triangle corresponds to a “double perovskite” composition Cs_2_CuInBr_6_. The synthesis has been performed at different temperatures to reach the most optimal thermodynamic parameters for the perovskite phase formation. Sintering at 300 °C leads to polycrystalline products, which are orange-colored powders. The samples annealed at 650 °C correspond to melt crystallization and are “single-piece” and orange-colored. No Cu^2+^ compounds are included as admixtures according to the ESR results of the samples.

The XRD data of the composition ‘1’ of the “double perovskite” Cs_2_CuInBr_6_ (Material Project ID: mp-1113481). The samples are not a single phase and correspond to a mixture of binary bromides, namely, cesium bromocuprate(I) and tricesium dibromoindate(III). Some reflections at the same 2Θ as for double halides, namely, CsCu_2_Br_3_ (PDF2 (38-850)) and Cs_3_In_2_Br_9_ (Springer materials ID: sd_1712349). 

In the ternary diagram, the “double perovskite” phase belongs to three double sections, including CuBr–Cs_2_InBr_5_, InBr_3_–Cs_2_CuBr_3_, and CsCu_2_Br_3_-Cs_3_In_2_Br_9_, respectively.

**Figure 2:**
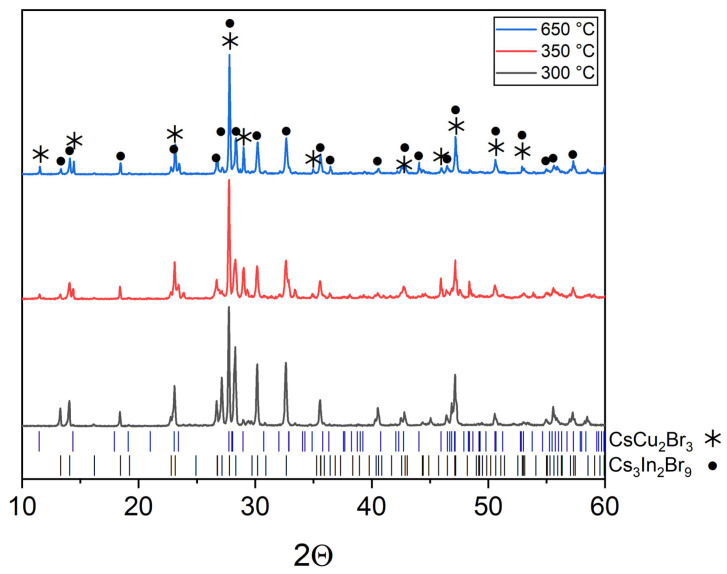
The diffraction pattern for the theoretical composition Cs_2_CuInBr_6_.

According to the phase, XRD analysis performed (Figure 2), the Cs_2_CuInBr_6_ phase is not formed in the whole temperature range of 300–650 °C, but the sample ‘1’ includes two phases of binary bromides Cs_3_In_2_Br_9_ and CsCu_2_Br_3_. No single bromides have been found as a result of the full transformation to binary bromides Cs_3_In_2_Br_9_ and CsCu_2_Br_3_. It is also noticeable that the XRD patterns for the samples annealed at 350 °C and 650 °C are much more similar than for the samples annealed at 300 °C and 350 °C, respectively. Most likely, the eutectic temperature for the composition ‘1’ is in the temperature range of 300–350 °C.

**Figure 3:**
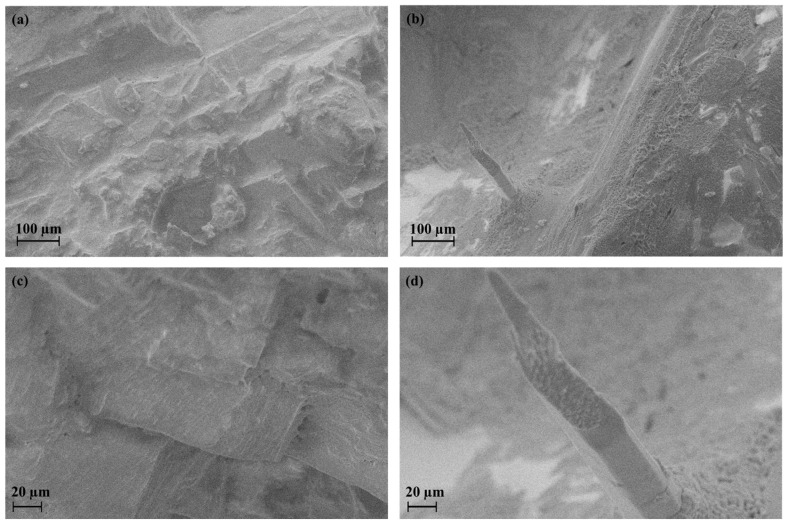
SEM images of composition ‘1’ obtained by the ampoule method at (**a**,**c**) 300 °C (**b**,**d**) 650 °C.

Figure 3 shows the micromorphology of the samples synthesized at 300 °C and 650 °C, respectively. The composition ‘1’ annealed at 300 °C is rather uniform and is presented by layered orthorhombic crystals. The composition ‘1’ annealed at 650 °C has a different micromorphology demonstrating the crystallization of the Cs_3_In_2_Br_9_ phase as prismatic crystals surrounded by a euthectic mixture of uniform round-shaped grains of 1–2 µm. It is evident that the eutectic point is closer to bromocuprate(I) cesium composition because of its lower melting point in comparison to tricesium dibromoindate(III). The composition ‘1’ melted definitely at 650 °C gives larger crystals of Cs_3_In_2_Br_9_ in a matrix of eutectic mixture of smaller crystals. 

**Figure 4:**
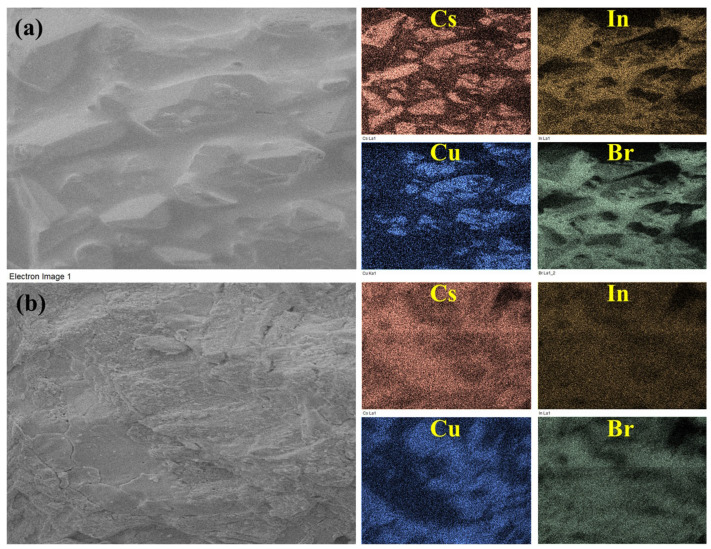
Element distribution map of composition ‘1’ obtained by the ampoule method at (**a**) 300 °C (**b**) 650 °C.

In order to refine the stoichiometry in the course of ampoule synthesis, some of the samples have been additionally studied by the EDS method. Table 2 and Figure 4 show the results of EDS spectroscopy of samples synthesized at 300 °C and 650 °C, respectively. It is shown that the cation ratio of Cu/In is close to 1:1. It can be assumed that minor deviations from theoretical values can be associated with different morphology of the crystallites of the resulting phases. According to EDS imaging (Figure 4), the composition ‘1’ after a sintering at 300 °C includes prismatic crystallites of complex bromide of copper and cesium. This correlates with XRD data and could be related to CsCu_2_Br_3_ phase.

**Figure 5:**
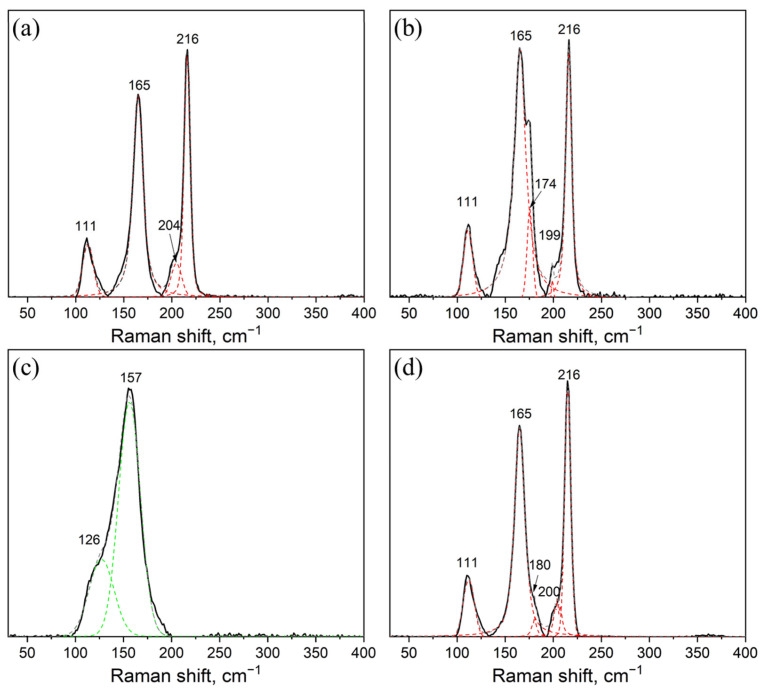
Raman spectra of samples of the theoretical composition Cs_2_CuInBr_6_ recently obtained by the ampoule method (**a**) ‘1’ at 300 °C (**b**) ‘1’ at 650 °C, (**c**) CsCu_2_Br_3_ and (**d**) Cs_3_In_2_Br_9_. Laser excitation 532 nm.

The Raman spectra of the samples synthesized at 300 °C and 650 °C are given in Figure 5a,b. The spectra contain well-defined characteristic modes related mostly to the Cs_3_In_2_Br_9_ phase. The most intensive modes are: ν (A_1g_)—165 cm^−1^, ν(E_g_)—216 cm^−1^ and 111 cm^−1^ [40]. Most likely, this is the result of higher crystallinity (larger size of crystallites) for the Cs_3_In_2_Br_9_ phase. 

**Figure 6:**
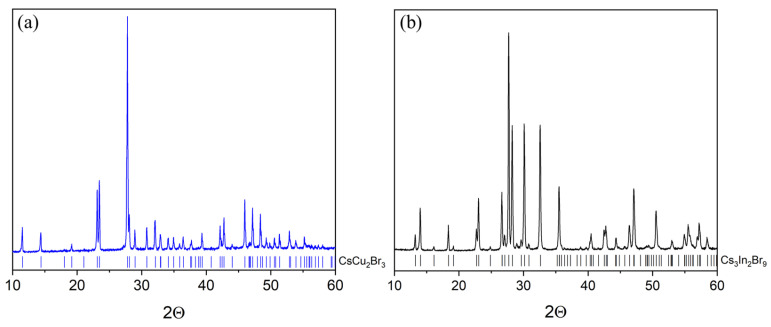
XRD data of (**a**) a sample ‘2’ (CsCu_2_Br_3_ phase), and (**b**) sample ‘3’ (Cs_3_In_2_Br_9_ phase).

A sample of the CsCu_2_Br_3_ (point ‘2’) binary bromide obtained at a temperature of 650 °C and an annealing time of 24 h is single-phase according to XRD data (Figure 6a). The sample Cs_3_In_2_Br_9_ (point ‘3’), synthesized at a temperature of 650 °C and an annealing time of 24 h, is obtained by crystallization from a melt. According to its XRD results, the sample is a single-phase and corresponds to the phase Cs_3_In_2_Br_9_ (Springer materials ID: sd_1712349). It’s identical to the sample synthesized by our group by the solid-phase method and reported elsewhere [41]. The Raman data for the sample (Figure 5d) also correspond well to the spectrum of the Cs_3_In_2_Br_9_ phase described by Zhou et al. [42] and later by Kamilov et al. [41].

Additional twelve compositions have been synthesized at the intersections of possible binary sections to analyze binary phase equilibria in the CsBr-CuBr-InBr_3_ ternary system. Namely, the intersection point of the CsCu_2_Br_3_-Cs_3_In_2_Br_9_ and CuBr-Cs_3_InBr_6_ sections (point ‘4’, obtained by displacement of bromides CsCu_2_Br_3_ and Cs_3_In_2_Br_9_), the CsCu_2_Br_3_-Cs_2_InBr_5_ and CuBr-Cs_3_InBr_6_ sections (point ‘5’), the Cs_2_CuBr_3_-InBr_3_ and CuBr-Cs_2_InBr_5_ (point ‘6’), the Cs_2_CuBr_3_-InBr_3_ and CsCu_2_Br_3_-Cs_2_InBr_5_ sections (point ‘7’), the Cs_2_CuBr_3_-Cs_3_In_2_Br_9_ and CuBr-Cs_3_InBr_6_ (point ‘8’), the Cs_2_CuBr_3_-Cs_3_In_2_Br_9_ and CsCu_2_Br_3_-Cs_2_InBr_5_ (point ‘9’), the Cs_2_CuBr_3_-Cs_3_In_2_Br_9_ and CuBr-Cs_2_InBr_5_ (point ‘10’), the CsCu_2_Br_3_-InBr_3_ and CuBr-Cs_3_InBr_6_ sections (point ‘12’), the CsCu_2_Br_3_-InBr_3_ and CuBr-Cs_2_InBr_5_ (point ‘13’), the CsCu_2_Br_3_-InBr_3_ and CuBr-Cs_3_In_2_Br_9_ (point ‘14’, obtained by displacement of bromides CuBr and Cs_3_In_2_Br_9_), the Cs_2_CuBr_3_-InBr_3_ and CuBr-Cs_3_In_2_Br_9_ (point ‘15’), and also point ‘11’ and point ‘16’ which are outside the intersections. The annealing temperature was 250–350 °C for points ‘8’ and ‘14’, 450 °C for point ‘11’, and 650 °C for points ‘4’–‘10’,’12’–’16’. 

**Figure 7:**
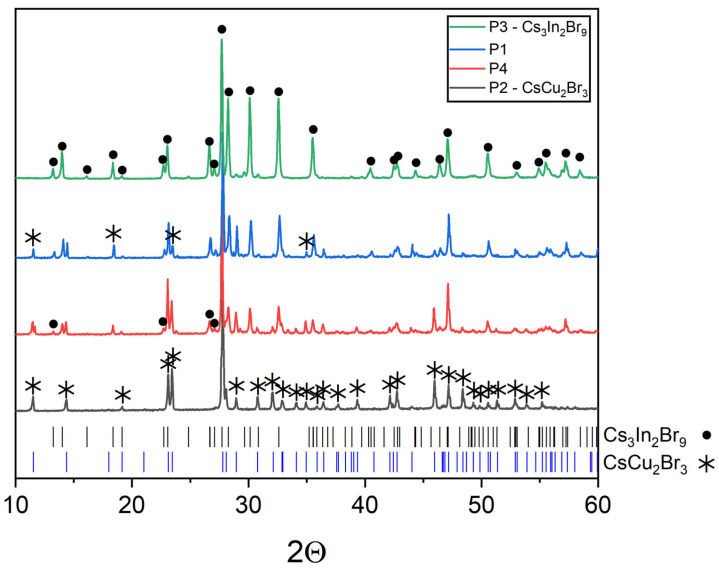
XRD data for the points ‘1–4’, annealed at 650 °C (incision CsCu_2_Br_3_-Cs_3_In_2_Br_9_).

The XRD data of points ‘1’–‘4’ located on the CsCu_2_Br_3_-Cs_3_In_2_Br_9_ binary section contain only reflections of the CsCu_2_Br_3_ (PDF (77-1586)) and Cs_3_In_2_Br_9_ (Springer materials ID: sd_1712349) phases that indicates the stability of this section (Figure 7). Sample ‘4’ (the intersection of the CsCu_2_Br_3_-Cs_3_In_2_Br_9_ and CuBr-Cs_3_InBr_6_ sections, obtained by displacement of bromides CsCu_2_Br_3_ and Cs_3_In_2_Br_9_ phases), annealed at 650 °C, does not have reflections of the CuBr and Cs_3_InBr_6_ phases, which may indicate the instability of the CuBr-Cs_3_InBr_6_ quasi-binary section. As a result, the simulated “double perovskite” phase Cs_2_CuInBr_6_ is in the quasi-binary section CsCu_2_Br_3_-Cs_3_In_2_Br_9_. No complex bromide compounds are predicted for this binary system. As it has been discussed above, the expected eutectic point is closer to the CsCu_2_Br_3_ phase because of its lower melting point. 

**Figure 8:**
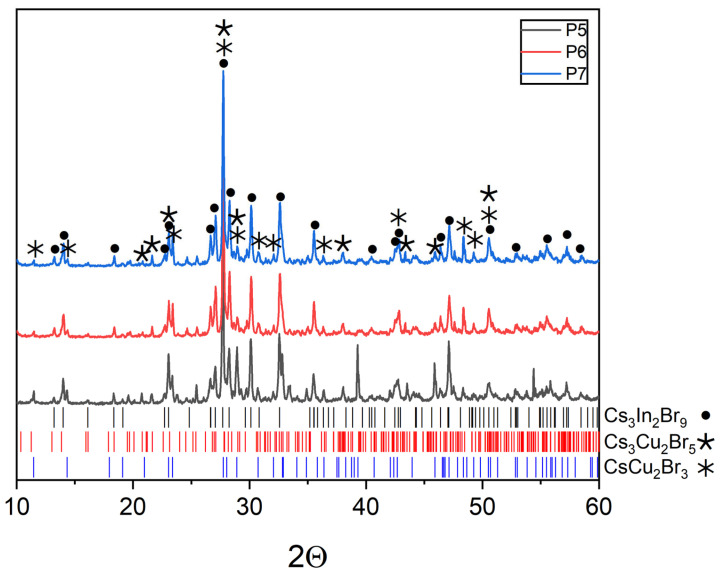
XRD data for point ‘5’ (intersection of incisions CsCu_2_Br_3_-Cs_2_InBr_5_ and CuBr-Cs_3_InBr_6_), point ‘6’ (intersection of incisions Cs_2_CuBr_3_-InBr_3_ and CuBr-Cs_2_InBr_5_), and point ‘7’ (intersection of incisions Cs_2_CuBr_3_-InBr_3_ and CsCu_2_Br_3_-Cs_2_InBr_5_).

**Figure 9:**
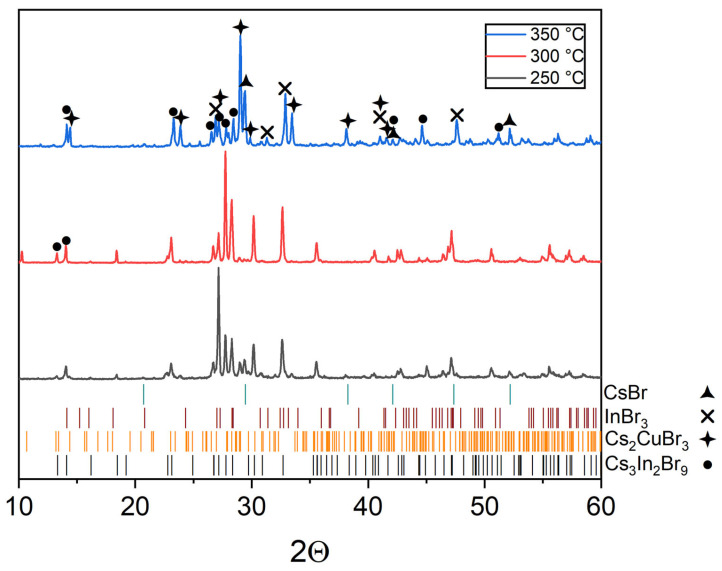
XRD data for point ‘8’ (intersection of incisions Cs_2_CuBr_3_-Cs_3_In_2_Br_9_ and CuBr-Cs_3_InBr_6_).

XRD of samples ‘5’, ‘6’, and ‘7’ are given in Figure 8. All three points are at the virtual intersections of the CsCu_2_Br_3_-Cs_2_InBr_5_ and CuBr-Cs_3_InBr_6_ sections (point ‘5’), the Cs_2_CuBr_3_-InBr_3_ and CuBr-Cs_2_InBr_5_ (point ‘6’), the Cs_2_CuBr_3_-InBr_3_ and CsCu_2_Br_3_-Cs_2_InBr_5_ sections (point ‘7’). The results show the instability of these intersections. Reflections of the most stable phases CsCu_2_Br_3_ (PDF2 (77-1586)), Cs_3_Cu_2_Br_5_ (PDF (01-072-9849)), and Cs_3_In_2_Br_9_ (Springer materials ID: sd_1712349) have been identified. Obviously, all three points are in a triangle of phases of binary bromides, namely, CsCu_2_Br_3_, Cs_3_Cu_2_Br_5,_ and Cs_3_Cu_2_Br_9_. Point ‘8’ (Figure 9) is on the intersection of incisions of CuBr-Cs_3_InBr_6_ and Cs_2_CuBr_3_-Cs_3_In_2_Br_9_. Equilibrium (2) is assumed to exist:

3Cs_2_CuBr_3_ + Cs_3_In_2_Br_9_ = 6CuBr + 2Cs_3_InBr_6_ (2)

According to the phase analysis performed, sample ‘8’ synthesized from the simple bromides contains the phases Cs_3_In_2_Br_9_ (Springer materials ID: sd_1712349), Cs_2_CuBr_3_ (Maretial Project ID: mp-1226699), InBr_3_ (PDF2 (79-281)), CsBr (PDF2 (78-615)). Thus, the stability of these four phases under synthesis conditions is shown. Cs_3_InBr_6_ phase shown in the phase diagram CsBr–InBr_3_ is not stable if performed a solid-state synthesis or crystallized from the melt. Thus, we can conclude that the Cs_3_InBr_6_-CuBr section is not quasi-binary.

**Figure 10:**
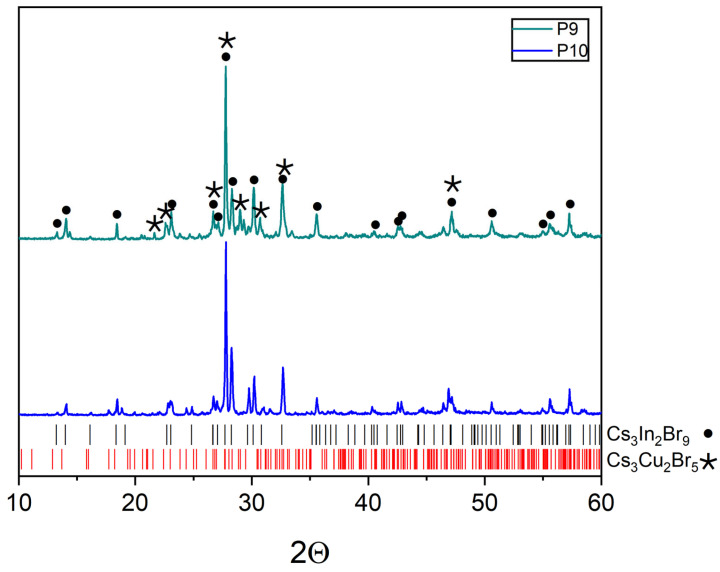
XRD data for point ‘9’ (intersection of incisions Cs_2_CuBr_3_-Cs_3_In_2_Br_9_ and CsCu_2_Br_3_-Cs_2_InBr_5_) and point ‘10’ (intersection of incisions Cs_2_CuBr_3_-Cs_3_In_2_Br_9_ and CuBr-Cs_2_InBr_5_).

Points ‘8’, ‘9’, and ‘10’ are on the inclusion Cs_2_CuBr_3_-Cs_3_In_2_Br_9_ section (Figure 1a). Points ‘9’ and ‘10’ contain phases Cs_3_In_2_Br_9_ (Springer materials ID: sd_1712349) and Cs_3_Cu_2_Br_5_ (PDF (01-072-9849)) according to the XRD results (Figure 10). Apparently, during crystallization from the melt on the Cs_2_CuBr_3_-Cs_3_In_2_Br_9_ section, the formation of the Cs_3_Cu_2_Br_5_ phase is preferable, while solid-phase synthesis leads to Cs_2_CuBr_3_.

**Figure 11:**
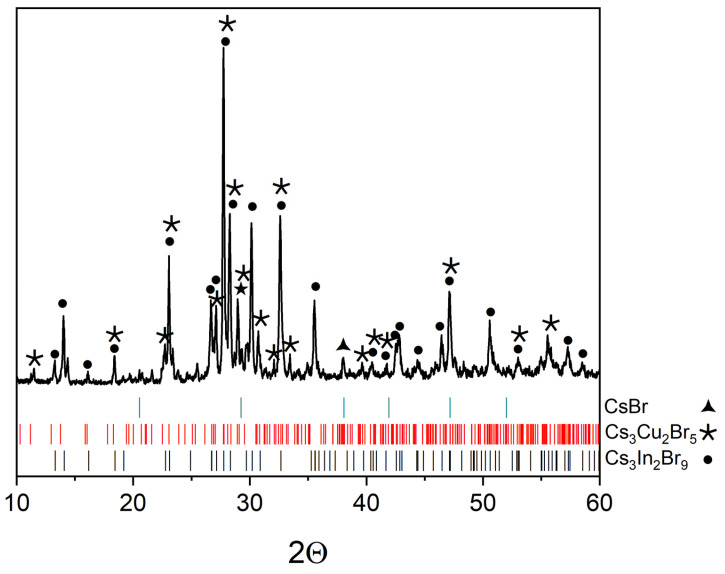
XRD results for point ‘11’.

The XRD data of point ‘11’, which is outside the intersections and located in a triangle of CsBr–Cs_2_CuBr_3_–Cs_3_InBr_6_ phases (Figure 1), is shown in Figure 11. According to the XRD data, reflections of the CsBr (PDF2 (78-615)), Cs_3_Cu_2_Br_5_ (PDF (01-072-9849)), and Cs_3_In_2_Br_9_ (Springer materials ID: sd_1712349) phases are found. Apparently, the Cs_3_InBr_6_, Cs_2_InBr_5_, and Cs_2_CuBr_3_ phases are not stable at 450 °C, which is consistent with the data on the CsBr–CuBr [36] and CsBr–InBr_3_ [39] binary systems.

**Figure 12:**
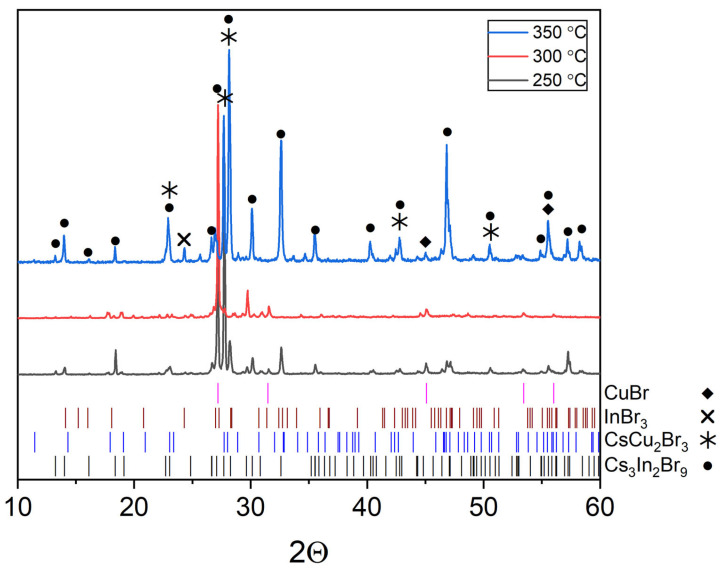
XRD results for point ‘14’ (intersection of incisions CsCu_2_Br_3_-InBr_3_ and CuBr-Cs_3_In_2_Br_9_).

The XRD patterns of sample ‘14’ is on the intersection of incisions of CsCu_2_Br_3_-InBr_3_ and CuBr-Cs_3_In_2_Br_9_ are given in Figure 12. Reflection identification shows that the samples are not single-phase as expected. The diffractograms show the presence of reflections of complex bromides, namely, as CsCu_2_Br_3_ (PDF-2 (77-1586)), Cs_3_In_2_Br_9_ (Springer materials ID: sd_1712349), as well as a precursor CuBr (PDF-2 (6-292)) and InBr_3_ (PDF-2 (79-281)). 

It can be assumed that there is an equilibrium described by Equation (3):

6CuBr + Cs_3_In_2_Br_9_ = 3CsCu_2_Br_3_ + 2InBr_3_ (3)

According to the experimental data, it is difficult to draw an unambiguous conclusion about the effect of temperature increase on the shift of equilibrium to the right or to the left since the number of reflections increases with a growth in the annealing temperature. The relative intensity of reflections changes, and reflections overlap each other. At the same time, based on single reflections, it can be seen that the relative intensity of the CuBr phase reflections decreases while the intensity of the main reflections of the CsCu_2_Br_3_, Cs_3_In_2_Br_9_, and InBr_3_ phases increases. Thus, it is most likely that equilibrium (3) shifts to the right with an increase in the annealing temperature. In this case, the increase in the intensity of reflections of the cesium bromoindate(III) phase can be explained by the formation of larger crystallites of this phase with increasing temperature.

**Figure 13:**
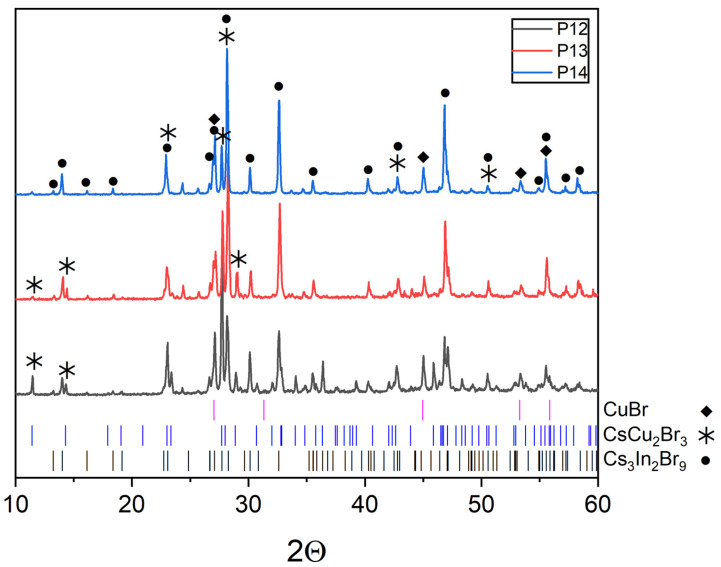
XRD data for point ‘12’ (intersection of incisions CsCu_2_Br_3_-InBr_3_ and CuBr-Cs_3_InBr_6_), point ‘13’ (intersection of incisions CsCu_2_Br_3_-InBr_3_ and CuBr-Cs_2_InBr_5_) and point ‘14’ (intersection of incisions CsCu_2_Br_3_-InBr_3_ and CuBr-Cs_3_In_2_Br_9_).

Points ‘12’, ‘13’, ‘14’ (Figure 1) are located at the intersection of the CsCu_2_Br_3_-InBr_3_ section and the CuBr-Cs_3_InBr_6_, CuBr-Cs_2_InBr_5_, CuBr-Cs_3_In_2_Br_9_ sections, respectively. According to XRD data (Figure 12), the phase composition of these points includes the CuBr (PDF2 (6-292)), CsCu_2_Br_3_ (PDF-2 (77-1586)), and Cs_3_In_2_Br_9_ (Springer materials ID: sd_1712349) phases. On the other hand, the phase composition of point ‘15’ according to XRD data (Figure 13), consists of the phase CuBr, CsCu_2_Br_3_, and Cs_3_In_2_Br_9_, as well as for points ‘12’, ‘13’, ‘14’. Summarizing these results, we can conclude that points ‘12’–’15’ lie in a triangle consisting of CuBr, CsCu_2_Br_3_, and Cs_3_In_2_Br_9_ phases (Figure 1), which corresponds to the phase composition of all four points. 

**Figure 14:**
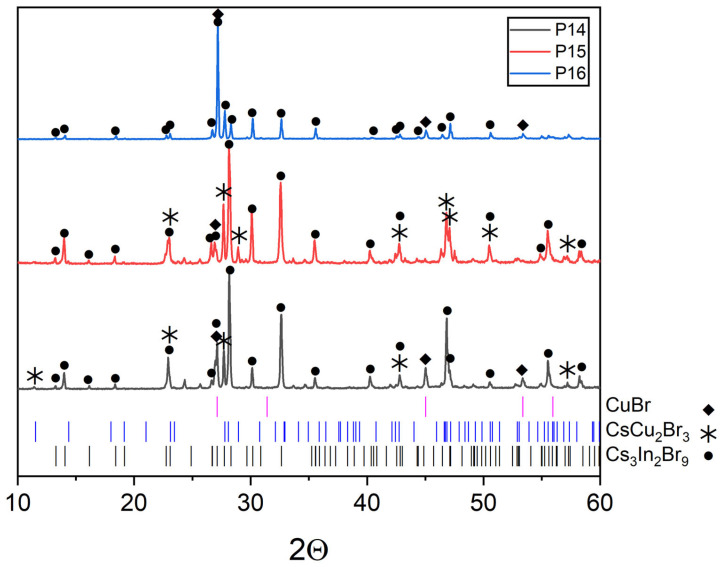
XRD patterns for the point ‘14’ (intersection of incisions CsCu_2_Br_3_-InBr_3_ and CuBr-Cs_3_In_2_Br_9_).

Also, the phase composition of point ‘16’ consists of the CuBr and Cs_3_Cu_2_Br_9_ phases due to the XRD data (Figure 14). This fact confirms the stability of the CuBr-Cs_3_In_2_Br_9_ section, point ‘15’ (intersection of incisions CuBr-Cs_3_In_2_Br_9_ and Cs_2_CuBr_3_-InBr_3_), point ‘16’.

The deepest minimum in the heating DSC curve is observed at 330 °C (603 K). This temperature is lower than the melting point of the CsCu_2_Br_3_ phase (congruent melting at 351 °C (624 K) [42]. There is no Cs_3_In_2_Br_9_ compound in the known phase diagram [43], and there are no data on the exact melting point of the Cs_3_In_2_Br_9_ phase. At higher temperatures, features on the DSC curve for this composition do not exist up to a temperature of 450 °C. It can be assumed that the temperature corresponds to the liquidus in the binary system CsCu_2_Br_3_-Cs_3_In_2_Br_9_. A relatively small minimum at 309 °C (582 K) may correspond to a phase transition for one of the components of the system (possibly Cs_3_In_2_Br_9_) or to a solidus isotherm in the quasi-binary system CsCu_2_Br_3_-Cs_3_In_2_Br_9_.

The cooling curve of sample ‘4’ has two maxima. The first (right) one, at 325 °C (598 K), is wider, less intense, and probably corresponds to the beginning of the crystallization process of Cs_3_In_2_Br_9_. The next (left) one at 294 °C (567 K), more intense, corresponds to the solidus isotherm for the quasi-binary system CsCu_2_Br_3_-Cs_3_In_2_Br_9_ and the end of crystallization process (probably, crystallization of the eutectic composition). According to the XRD results, the phase composition of sample ‘4’ has not changed.

**Figure 15:**
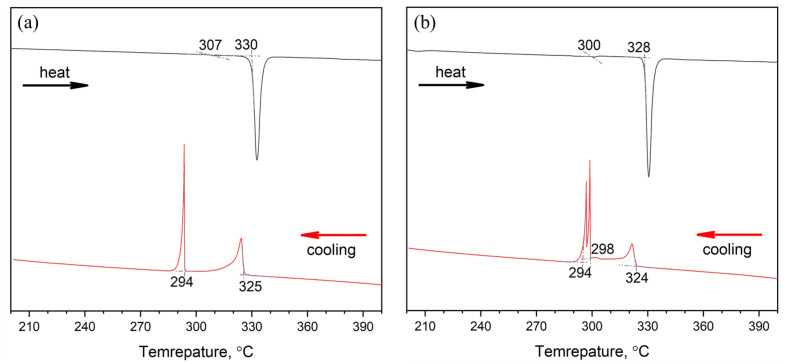
DSC data for the composition of (**a**) point ‘4’ at the intersection of the sections CuBr-Cs_3_InBr_6_, CsCu_2_Br_3_-Cs_3_In_2_Br_9_ synthesized from bromides CuBr and Cs_3_In_2_Br_9_ and (**b**) point ‘14’ at the intersection of the sections CuBr-Cs_3_In_2_Br_9,_ CsCu_2_Br_3_-InBr_3_ obtained by displacement of binary bromides CsCu_2_Br_3_ and Cs_3_In_2_Br_9_. Black line is heating, and red line is cooling.

The heating DSC curve for a batch sample containing CuBr and Cs_3_In_2_Br_9_ in stoichiometric amounts corresponding to composition ‘14’ is shown in Figure 15b. A very weak endothermic effect is found at 300 °C (573 K). The deepest minimum on the melting curve is observed at 328 °C (601 K) and, most likely, corresponds to a liquidus temperature for the given composition. At higher temperatures (up to 450 °C), no other endothermic effects are observed. There are three exothermic maxima on the cooling curve, the profile, two of which repeat the results for the heating curve of the two-phase system CsCu_2_Br_3_-Cs_3_In_2_Br_9_. 

A new sharp crystallization maximum also appears on the cooling curve at 298 °C (571 K), which can be explained by the crystallization of the third phase, which may be InBr_3_ or CsCu_2_Br_3_. The appearance of a new crystallization maximum can be associated with the solid-state process described by Equation (2) above. The narrow maximum at 294 °C (567 K) could be a solidus temperature for the quasi-binary system of CuBr-Cs_3_In_2_Br_9_ or, probably, CsCu_2_Br_3_-Cs_3_In_2_Br_9_ (see above for the reasons). The DSC data for the composition ‘14’ are in good agreement with the XRD results reported for this composition above. 

## 4. Conclusions

Investigation of the phase equilibria discussed in the article is rather rough but gives important information about the processes which take part in the ternary systems on heating. The predicted theoretically bromindate(III) Cs_2_CuInBr_6_ with a double perovskite structure has not been obtained yet using a direct synthesis from primary or double bromides as precursors, even under a rather long sintering process. The reason for this instability of the phase originates from the high stability of corresponding binary bromides Cs_3_In_2_Br_9_ and CsCu_2_Br_3_.

At the same time, the current research sheds light on the phase composition and chemical processes in the phase triangle of bromides CsBr-CuBr-InBr_3_. The quasi-binarity of the CsCu_2_Br_3_-Cs_3_In_2_Br_9_ and Cs_2_CuBr_3_-Cs_3_In_2_Br_9_ section is experimentally shown under the conditions of solid-phase synthesis. A possibility of the solid-phase reaction Cs_3_In_2_Br_9_ + CuBr = CsCu_2_Br_3_ + InBr_3_ is shown by the method of ampoule synthesis. As the temperature rises, the reaction equilibrium shifts to the right. It is shown that during crystallization from the melt on the Cs_2_CuBr_3_-Cs_3_In_2_Br_9_ section, the formation of the Cs_3_In_2_Br_9_ and Cs_3_Cu_2_Br_5_ phases is preferable. Thereby, the CsBr-CuBr-InBr_3_ ternary system triangulates with the next eutectic-type quasi-binary sections Cs_3_Cu_2_Br_5_-Cs_3_In_2_Br_9_, CsCu_2_Br_3_-Cs_3_In_2_Br_9_, and CuBr-Cs_3_In_2_Br_9_, forming four triangles CsBr-Cs_3_Cu_2_Br_5_-Cs_3_In_2_Br_9_, CsCu_2_Br_3_-Cs_3_Cu_2_Br_5_-Cs_3_In_2_Br_9_, CuBr-CsCu_2_Br_3_-Cs_3_In_2_Br_9_, CuBr-Cs_3_In_2_Br_9_-InBr_3_.

## Figures and Tables

**Table 1:** Weighed weights of CsBr, CuBr, and InBr_3_ simple bromides for the samples, calculated per 1 g of the final product.

Sample	Mole Fraction of Precursors			Weight of Precursors per 1 g of Product		
	n(CsBr)	n(CuBr)	n(InBr_3_)	m(CsBr)	m(CuBr)	m(InBr_3_)
Point 1	0.500	0.250	0.250	0.461(1)	0.155(1)	0.384(1)
Point 2	0.330	0.660	-	0.426(1)	0.572(2)	-
Point 3	0.600	-	0.400	0.474(1)	-	0.526(1)
Point 4	0.427	0.431	0.142	0.448(1)	0.305(1)	0.248(1)
Point 5	0.499	0.335	0.166	0.499(2)	0.226(1)	0.276(2)
Point 6	0.533	0.267	0.200	0.510(2)	0.172(1)	0.318(2)
Point 7	0.545	0.274	0.181	0.529(3)	0.179(1)	0.293(1)
Point 8	0.631	0.159	0.210	0.580(2)	0.099(1)	0.322(1)
Point 9	0.618	0.096	0.286	0.533(3)	0.056(1)	0.411(1)
Point 10	0.614	0.077	0.309	0.520(1)	0.044(1)	0.436(2)
Point 11	0.800	0.100	0.100	0.774(3)	0.065(1)	0.161(2)
Point 12	0.300	0.600	0.100	0.344(1)	0.465(2)	0.191(1)
Point 13	0.285	0.571	0.144	0.313(2)	0.424(1)	0.263(1)
Point 14	0.272	0.544	0.184	0.288(1)	0.388(1)	0.324(1)
Point 15	0.459	0.231	0.310	0.406(1)	0.138(1)	0.456(2)
Point 16	0.300	0.350	0.350	0.268(4)	0.211(1)	0.521(3)

**Table 2:** The atomic percentages of the elemental composition of the samples obtained by the ampoule method at T = 300 °C and 650 °C.

Synthesis Temperature, °C	Cs	Cu	In	Br	Cation Ratio Cu/In
T = 300 °C (solid-phase synthesis)	25	12	11	52	1.08
T = 650 °C (crystallization from the melt)	25	11	11	53	1.00

## Data Availability

Not applicable.
